# Progress of Total Abstinence

**Published:** 1842-05

**Authors:** 


### PROGRESS OF TOTAL ABSTINENCE.

We have lately made personal observations on the progress of the Temperance Reform from St. Louis, Missouri, to Chillicothe, Ohio, and are enabled to state that an extraordinary reduction in the use of intoxicating drinks has taken place within the last six months. Temperate drinkers have ceased to indulge themselves, and a vast number of habitual drunkards have suddenly broken off, and joined in the crusade against the use of alcohol. No class of men should experience more gratification at this reform than physicians, but we should not content ourselves with the happiness common to all benevolent men, when the sudden abandonment of a habit which all the world has regarded as pernicious to health, affords an admirable opportunity of studying its effects. We would then seriously urge on our readers, first, to note carefully the effects on health and constitution, of a sudden discontinuance of alcoholic drinks, previously taken to excess; second, to observe hereafter what diseases may disappear, in consequence of the diminution in the consumption of strong drinks.

